# Exact Renormalization Groups As a Form of Entropic Dynamics

**DOI:** 10.3390/e20010025

**Published:** 2018-01-04

**Authors:** Pedro Pessoa, Ariel Caticha

**Affiliations:** Department of Physics, University at Albany–SUNY, Albany, NY 12222, USA

**Keywords:** maximum entropy, exact renormalization group, entropic dynamics, entropic inference, renormalization

## Abstract

The Renormalization Group (RG) is a set of methods that have been instrumental in tackling problems involving an infinite number of degrees of freedom, such as, for example, in quantum field theory and critical phenomena. What all these methods have in common—which is what explains their success—is that they allow a systematic search for those degrees of freedom that happen to be relevant to the phenomena in question. In the standard approaches the RG transformations are implemented by either coarse graining or through a change of variables. When these transformations are infinitesimal, the formalism can be described as a continuous dynamical flow in a fictitious time parameter. It is generally the case that these exact RG equations are functional diffusion equations. In this paper we show that the exact RG equations can be derived using entropic methods. The RG flow is then described as a form of entropic dynamics of field configurations. Although equivalent to other versions of the RG, in this approach the RG transformations receive a purely inferential interpretation that establishes a clear link to information theory.

## 1. Introduction

The Renormalization Group (RG) is a collection of techniques designed for tackling problems that involve an infinite number of coupled degrees of freedom. The range of applications is enormous, it includes quantum field theory, the statistical mechanics of critical phenomena, turbulence, and many others. Ever since the work of Wilson (see, e.g., [1,2]) it has been clear that the various RGs succeed because they provide a systematic procedure to construct an effective theory for those variables that are most relevant to the problem at hand. For example, in Wilson’s approach to critical phenomena the procedure consists in gradually integrating out the degrees of freedom with short wavelengths to obtain an effective Hamiltonian for the long wavelengths that are empirically relevant [1].

The RG transformations are implemented by either eliminating degrees of freedom through coarse graining, through a change of variables, or by a combination of the two. The result is that the RG transformations generate a continuous flow in the statistical manifold of Gibbs distributions. One crucial early insight [1,3] was that infinitesimal RG transformations could be implemented exactly. (This formalism is now variously known as the exact RG, the functional RG, and the non-perturbative RG). This has both conceptual and computational advantages. On the conceptual side, for example, the work of Polchinski [4] used an exact RG as a method to prove renormalizability. On the computational side, the exact RG was extensively exploited by C. Wetterich [5,6] and coauthors, the effective average action method, in statistical mechanics [7] and also in Yang-Mills theory [8] and gravity [9]. More recently, also on the computational side, the work of N. Caticha and collaborators points in the direction of deploying RG techniques for data analysis [10].

Another crucial contribution was Wegner’s realization that the elimination of degrees of freedom is not strictly necessary, that an appropriate change of variables could effectively accomplish the same task [11]. The precise form of those changes of variables have been elaborated by a number of authors [12,13,14,15]. In [12] the reason why RGs are useful is particularly clear: the changes of variables are such that a classical or saddle-point approximation becomes more accurate, asymptotically approaching the exact result, and therefore offering a way to reach beyond the limitations of perturbation theory. (For additional references see the excellent reviews [16,17,18,19,20,21].)

In this paper we develop a new approach to the exact RG, derived as an application of entropic methods of inference—and entropic renormalization group. (The principle of maximum entropy as a method for inference can be traced to the pioneering work of Jaynes [22,23,24]. For a pedagogical overview of Bayesian and entropic inference and further references see [25].) The motivation is two-fold. First, although it is equivalent to other versions of the exact RG, in this approach the RG transformations receive a purely inferential interpretation that establishes a clear link to information theory. Second, it turns out that the RG flow is described as a form of Entropic Dynamics (ED). ED had previously been deployed to derive quantum theory as a form of inference both for particles (see, e.g., [26,27]) and for fields [28]. The formulation of an ED version of RG presented here is a first step towards establishing a closer link between RG techniques to the very foundations of quantum field theory. The natural expectation is that this will lead to further insights into Yang-Mills and gravity theory.

In Section 2 and Section 3, we establish notation and give a brief review of the RG as an exact change of variables. The derivation of the RG as a form of Entropic Dynamics is given in Section 4.

## 2. Some Background and Notation

Our subject is the statistical mechanics of a scalar field $\phi \left(x\right)=\phi_{x}$ in *d* spatial dimensions; such a field configuration can be represented as a point ϕ in an *∞*-dimensional configuration space C. The Fourier components are denoted

(1) $$\phi_{q}=\int dx\;\phi_{x}e^{iq\cdot x}\;,$$

where $dx=d^{d}x$. In thermal equilibrium the probability distribution of $\phi_{x}$ is of the Gibbs form,

(2) $$\rho \left[\phi \right]=\frac{1}{Z}e^{-H[\phi ]}\;\mathrm{where}\;Z=\int \left(\prod_{q}d\phi_{q}\right)\;e^{-H[\phi ]}$$

is the partition function, and a factor β=1/kT has been absorbed into the Hamiltonian *H*.

In this section, for simplicity, we describe the paradigmatic example of a sharp-cutoff RG. The sequence of RG transformations generates a trajectory of effective Hamiltonians $H_{\tau }$ labeled by a parameter τ. Suppose the integration over all $\phi_{q}$s with *q* higher than a certain cutoff Λ has been performed. Then the partition function takes the form

(3) $$Z=\int \left(\prod_{q<\Lambda }d\phi_{q}\right)\;e^{-H_{\tau }\left[\phi \right]}\;\mathrm{where}\;e^{-H_{\tau }\left[\phi \right]}=\int \left(\prod_{q>\Lambda }d\phi_{q}\right)\;e^{-H[\phi ]}\;.$$

The infinitesimal RG transformation requires two steps. The first involves integrating out those wavelengths in the narrow shell with $\Lambda e^{-\delta \tau }<q<\Lambda$ leading to

(4) $$Z=\int \left(\prod_{q<\Lambda e^{-\delta \tau }}d\phi_{q}\right)\;e^{-H_{\tau +\delta \tau }^{\prime }\left[\phi \right]}\;,$$

where

(5) $$e^{-H_{\tau +\delta \tau }^{\prime }\left[\phi \right]}=\int \left(\prod_{\Lambda e^{-\delta \tau }<q<\Lambda }d\phi_{q}\right)\;e^{-H_{\tau }\left[\phi \right]}\;.$$

Since this is an infinitesimal transformation it can be carried out exactly [3,12]. The result is

(6) $$H_{\tau +\delta \tau }^{\prime }-H_{\tau }={\left(2\pi \right)}^{d}\Lambda^{d-2}d\tau \int d\Omega_{d}\left[\frac{\delta^{2}H_{\tau }}{\delta \phi_{q}\delta \phi_{-q}}-\frac{\delta H_{\tau }}{\delta \phi_{q}}\frac{\delta H_{\tau }}{\delta \phi_{-q}}\right]$$

where $q^{2}=\Lambda^{2}$ and $d\Omega_{d}\;$ is the element of solid angle in *d* dimensions. The typical RG transformation includes a second step in which momenta and fields suitably re-scaled to yield $H_{\tau +\delta \tau }$. The momenta are scaled by $q\to qe^{\delta \tau }$ so that throughout the RG flow the new momenta always span the same constant range (0,Λ). The rescaling of the fields is

(7) $$\phi_{x}\to \phi_{x^{\prime }}^{\prime }=e^{d_{\phi }\delta \tau }\phi_{x}\;\mathrm{or}\;\phi_{q}\to \phi_{q^{\prime }}^{\prime }=e^{(d_{\phi }-d)\delta \tau }\phi_{q}\;,$$

where the field scale dimension $d_{\phi }=d/2-1+\gamma_{\phi }$ includes the $\gamma_{\phi }$ correction—the anomalous dimension—needed for the trajectory to flow towards a fixed point $H_{\infty }$ as τ→∞.

## 3. The RG As a Change of Variables

One advantage of expressing the partition function as an integral is that we can easily study the effects induced by transformations of the dynamical variables. This allows us to explore the idea that the RG is a technique that selects the relevant variables as they transform through different scales. Generalizing beyond the sharp cutoff case discussed in the previous section, the partition function at some stage τ of the RG flow can, in general, be written as

(8) $$Z=\int D\phi \;e^{-H_{\tau }\left[\phi \right]}\;\mathrm{where}\;D\phi =\prod_{q}d\phi_{q}\;,$$

with no limitations on the range of q. As τ→−∞, the effective Hamiltonian tends to the bare Hamiltonian in (2), $H_{\tau }\to H_{-\infty }=H$. Consider an infinitesimal change of variables,

(9) $$\phi_{q}\to \phi_{q}^{\prime }=\phi_{q}-\delta \tau \;\eta_{\tau q}\left[\phi \right]\;,$$

where $\eta_{\tau q}\left[\phi \right]$ is some sufficiently well-behaved functional of ϕ and a function of *q*. Then Equation (8) becomes

(10) $$Z=\int D\phi \;\left[1-\delta \tau \int dq\frac{\delta \eta_{\tau q}\left[\phi \right]}{\delta \phi_{q}}\right]\exp-\left[H_{\tau }\left[\phi \right]-\delta \tau \int dq\frac{\delta H_{\tau }}{\delta \phi_{q}}\eta_{\tau q}\left[\phi \right]\right]\;,$$

where $dq=d^{d}q/{\left(2\pi \right)}^{d}$. This leads to

(11) $$Z=\int D\phi \;\exp-H_{\tau +\delta \tau }\left[\phi \right]\;,$$

where

(12) $$H_{\tau +\delta \tau }\left[\phi \right]=H_{\tau }\left[\phi \right]-\delta \tau \int dq\left[\frac{\delta H_{\tau }}{\delta \phi_{q}}\eta_{\tau q}\left[\phi \right]-\frac{\delta \eta_{\tau q}\left[\phi \right]}{\delta \phi_{q}}\right]\;.$$

As discussed in [12], the choice of $\eta_{\tau }$ that reproduces an RG transformation (see, e.g., Equation (6)) is $\eta_{\tau q}\left[\phi \right]∼\delta H_{\tau }/\delta \phi_{-q}$. The effect of integrating out short wavelengths as opposed to long wavelengths is achieved by an appropriate *q*-dependent proportionality constant $f_{q}$, which plays the role of a cut-off function. Typically we want some positive $f_{q}$ that leaves long wavelengths unmodified while effectively integrating out the short wavelengths. A suitable choice is, for example, $f_{q}∼q^{2}/\Lambda^{2}$, so that $f_{q}$ is small for q≪Λ, and $f_{q}$ is large for q≫Λ, where Λ is some reference momentum. The complete RG transformation also involves an additional scaling of momenta $q\to qe^{\delta \tau }$ and fields, Equation (7). The full change of variables is

(13) $$\eta_{\tau q}=\;f_{q}\frac{\delta H_{\tau }}{\delta \phi_{-q}}+\zeta_{q}\phi_{q}\;\mathrm{where}\;\zeta_{q}=d-d_{\phi }+q\cdot \frac{\partial }{\partial q}\;.$$

The corresponding exact RG equation is

(14) $$\frac{\partial }{\partial \tau }H_{\tau }=\int d^{d}q\;\left[f_{q}\left(\frac{\delta^{2}H_{\tau }}{\delta \phi_{q}\delta \phi_{-q}}-\frac{\delta H_{\tau }}{\delta \phi_{q}}\frac{\delta H_{\tau }}{\delta \phi_{-q}}\right)+\frac{\delta H_{\tau }}{\delta \phi_{q}}\zeta_{q}\phi_{q}\right]\;.$$

It turns out that observable quantities such as critical exponents are independent of the particular choice of $f_{q}$. For later convenience we rewrite (12) as an equation for $\rho_{\tau }=e^{-H_{\tau }\left[\phi \right]}/Z$. The result is remarkably simple,

(15) $$\frac{\partial }{\partial \tau }\rho_{\tau }=-\int dq\;\frac{\delta }{\delta \phi_{q}}\left(\rho_{\tau }\eta_{\tau q}\right)\;.$$

## 4. The Entropic Renormalization Group

Next we derive the RG evolution as a form of entropic dynamics (for the related ED of *quantum* scalar fields see [28]). We consider a generic probability distribution $\rho_{\tau }\left[\phi \right]$ and we wish to study how it flows as a function of the parameter τ.

The basic “dynamical” assumption is that under the RG flow the fields follow continuous trajectories. This means that a finite transformation can be analyzed as a sequence of infinitesimally short steps and allows us to focus our attention on infinitesimal RG transformations.

Given that a certain field configuration ϕ is transformed into a neighboring one $\phi^{\prime }$, we ask, what can we expect $\phi^{\prime }$ to be? It is common practice to define a coarse graining transformation that allows one to calculate $\phi^{\prime }$ from the given ϕ. Such RGs lead to a deterministic dynamics. In contrast, the essence of an entropic dynamics is that the information about the new $\phi^{\prime }$ is very limited and the goal is to determine a transition probability $P[\phi^{\prime }|\phi ]$. Thus, the entropic RG leads to an inherently indeterminist dynamics.

The transition probability $P[\phi^{\prime }|\phi ]$ is assigned by maximizing the entropy,

(16) $$S\left[P;Q\right]=-\int D\phi^{\prime }\;P\left[\phi^{\prime }|\phi \right]\log\frac{P[\phi^{\prime }|\phi ]}{Q[\phi^{\prime }|\phi ]}\;,$$

relative to the prior $Q[\phi^{\prime }|\phi ]$, and subject to any further information in the form of constraints (the goal of maximum entropy is an inference technique to update from one distribution (the prior) to another distribution (the posterior) when information is provided in the form of constraints). The choice of the logarithmic entropy, as opposed to Renyi or Tsallis entropies, is significant. The RG is a method to predict the physical correlations between long wavelength fields; it is essential that the method of inference itself do not contaminate the analysis by introducing unwarranted correlations.

### 4.1. The Prior

We adopt a prior that incorporates the information that the fields change by infinitesimal amounts but is otherwise very uninformative. We want a prior that does not introduce unwarranted correlations while reflecting the basic rotational and translational symmetry of *d*-dimensional space—a field degree of freedom $\phi_{x}$ located at *x* is not in any way different from another $\phi_{x^{\prime }}$ at $x^{\prime }$. Such a prior is given by a product of Gaussians,

(17) $$Q\left(\phi^{\prime }|\phi \right)\propto \exp-\frac{1}{2\Delta \tau }\int dq\;\frac{1}{2f_{q}}\Delta \phi_{q}\Delta \phi_{-q}\;,$$

where $\Delta \phi_{q}=\phi_{q}^{\prime }-\phi_{q}$. The various factors of two are chosen for later convenience. (See Equation (28) below. The units of τ are such that the exponent in (17) is dimensionless.) The crucial factor $1/f_{q}$, see Equation (13), enforces a different treatment for different scales; it implements the basic idea that field components with long wavelengths remain unchanged. The limit of infinitesimally short steps will be eventually implemented by taking Δτ→0.

### 4.2. The Constraint

The possibility of directionality in the dynamical flow is introduced through a constraint involving a drift potential Ω[ϕ]. The constraint takes the form of the expected value of the change of Ω in the short step $\Delta \phi_{x}$,

(18) $${\langle \Delta \Omega \rangle }_{P}=\kappa \;.$$

The specific form of the drift potential Ω that implements the rescaling of fields, and the numerical value κ will be determined below. This constraint can be written as

(19) $${\langle \int dx\;\frac{\delta \Omega }{\delta \phi_{x}}\Delta \phi_{x}\rangle }_{P}=\int D\phi^{\prime }\;P\left[\phi^{\prime }|\phi \right]\left(\int dq\;\frac{\delta \Omega }{\delta \phi_{q}}\Delta \phi_{q}\right)=\kappa \;.$$

### 4.3. The Transition Probability

The distribution $P[\phi^{\prime }|\phi ]$ that maximizes S[P;Q] subject to (19) and normalization is

(20) $$P\left[\phi^{\prime }|\phi \right]\propto \exp-\int dq\;\left[\frac{1}{4f_{q}\Delta \tau }\Delta \phi_{q}\Delta \phi_{-q}-\frac{\delta \Omega }{\delta \phi_{q}}\Delta \phi_{q}\right]$$

where the Lagrange multiplier has been absorbed into Ω. The transition probability (20) is a Gaussian, more conveniently written as

(21) $$P\left[\phi^{\prime }|\phi \right]=\frac{1}{Z}\exp-\frac{1}{2\Delta \tau }\int dq\;\frac{1}{2f_{q}}\left(\Delta \phi_{q}-\frac{\delta \Omega }{\delta \phi_{-q}}2f_{q}\Delta \tau \right)\left(\Delta \phi_{-q}-\frac{\delta \Omega }{\delta \phi_{q}}2f_{q}\Delta \tau \right).$$

This ED is a standard Wiener process. A generic step can be written as the sum of a drift and a fluctuation, $\Delta \phi_{q}=\langle \Delta \phi_{q}\rangle +\Delta w_{q}$, such that

(22) $$\langle \Delta \phi_{q}\rangle =\frac{\delta \Omega }{\delta \phi_{-q}}2f_{q}\Delta \tau \;,\;\langle \Delta w_{q}\rangle =0\;,\;\mathrm{and}\;\langle \Delta w_{q}\Delta w_{-q^{\prime }}\rangle =2f_{q}\Delta \tau \;\delta_{qq^{\prime }}\;.$$

### 4.4. Entropic Dynamics in Integral Form

The dynamics induced by $P[\phi^{\prime }|\phi ]$ follows from the rules of probability theory applied to the joint probability of two successive configurations ϕ and $\phi^{\prime }$. Marginalizing $\rho [\phi^{\prime },\phi ]$,

(23) $$\int D\phi \;\rho \left[\phi^{\prime },\phi \right]=\int D\phi \;P\left[\phi^{\prime }|\phi \right]\rho_{\tau }\left[\phi \right]=\rho_{\tau +\delta \tau }\left[\phi^{\prime }\right]\;.$$

This is the ED equation of evolution. It describes a coarse-graining and a drift, but notice that what is being coarse-grained here is the distribution $\rho_{\tau }\left[\phi \right]$ and not the field configuration ϕ itself. Notice also that Equation (23) is of the form of a Chapman-Kolmogorov equation but there is a subtle difference in that Equation (23) is not meant to describe a Markovian process that occurs in an already existing “physical” background time. Here there is no pre-existing background time; the “RG time τ” is being created by the entropic dynamics itself in such a way that, given the “present” $\rho_{\tau }$, the “future” $\rho_{\tau +\delta \tau }$ is statistically independent of the “past” $\rho_{\tau -\delta \tau }$.

### 4.5. The Arrow of RG Time

Equation (23) is strongly directional: $\rho_{\tau }\left[\phi \right]$ is prior and $\rho_{\tau +\delta \tau }\left[\phi^{\prime }\right]$ is posterior. Applying the rules of ED to $\rho_{\tau +\delta \tau }\left[\phi^{\prime }\right]$ leads forward to $\rho_{\tau +2\delta \tau }\left[\phi^{\prime \prime }\right]$; they do not lead back to $\rho_{\tau }\left[\phi \right]$. Granted, the rules of probability theory also allow us to construct a time-reversed evolution,

(24) $$\int D\phi^{\prime }\;P\left[\phi |\phi^{\prime }\right]\rho_{\tau +\delta \tau }\left[\phi^{\prime }\right]=\rho_{\tau }\left[\phi \right]\;\;,$$

but $P[\phi |\phi^{\prime }]$ is a very different object related to $P[\phi^{\prime }|\phi ]$ by Bayes’ theorem,

(25) $$P\left[\phi |\phi^{\prime }\right]=\frac{\rho_{\tau }\left[\phi \right]}{\rho_{\tau +\delta \tau }\left[\phi^{\prime }\right]}P\left[\phi |\phi^{\prime }\right]\;.$$

Thus, the asymmetry between priors and posteriors leads to an asymmetry between the inferential past and the inferential future—if $P[\phi^{\prime }|\phi ]$ is a Gaussian derived from the maximum entropy method, then the time-reversed $P[\phi |\phi^{\prime }]$ is obtained from Bayes’ theorem and is not Gaussian in general.

### 4.6. Entropic Dynamics in Differential Form

The ED described by (23) can be written as a functional differential equation of the Fokker-Planck type,

(26) $$\frac{\partial }{\partial \tau }\rho_{\tau }=-\int dq\;\frac{\delta }{\delta \phi_{q}}\left(\rho_{\tau }v_{q}\right)\;,$$

where $v_{q}\left[\phi \right]$ is the *q*-component of the “current” velocity with which probabilities flow in the *∞*-dimensional space C. (For algebraic details in finite dimensions see ([25]). The combination $\int dq\;\delta /\delta \phi_{q}$ is the functional equivalent of the divergence operator.) The current velocity $v_{q}$ is the sum of two contributions, a drift and an osmotic component

(27) $$v_{q}\left[\phi \right]=b_{q}\left[\phi \right]+u_{q}\left[\phi \right]=2f_{q}\frac{\delta \Omega }{\delta \phi_{-q}}-f_{q}\frac{\delta \log\rho_{\tau }}{\delta \phi_{-q}}$$

where the first and second terms are respectively called the drift and osmotic velocities.

### 4.7. Equivalence with the RG Change of Variables

So far we discussed the ED evolution, Equation (26), of a generic distribution $\rho_{\tau }\left[\phi \right]$ in a fictitious time τ. To make contact with the RG evolution, we set $\rho_{\tau }=e^{-H_{\tau }\left[\phi \right]}/Z$ with initial condition $H_{\tau }\to H$ (the bare Hamiltonian) as τ→−∞, and with *Z* independent of τ. Then the current velocity (27) is

(28) $$v_{q}=f_{q}\frac{\delta H_{\tau }}{\delta \phi_{-q}}+2f_{q}\frac{\delta \Omega }{\delta \phi_{-q}}\;.$$

Comparing Equation (26) with (15), which amounts to comparing (28) with (13), shows that the ED evolution is identical with the RG evolution provided we choose a drift potential Ω such that

(29) $$2f_{q}\frac{\delta \Omega }{\delta \phi_{-q}}=\zeta_{q}\phi_{q}\;.$$

The solution to this functional differential equation for Ω[ϕ] is some functional that is quadratic and possibly non-local in the fields. Fortunately, however, an explicit solution is not needed. None of the basic ED equations: the constraint (19), the transition probability (21), and the RG equation (26) with (28), require knowledge of Ω; we only need to know its gradient, Equation (29).

## 5. Final Remarks

To summarize our conclusions: the evolution of probability distributions under exact RG transformations can be formulated as a form of entropic dynamics. This establishes a clear link between the RG and information theory. This is not totally unexpected since the goal of the RG method is to select variables that best capture the relevant information about long distance behavior, while on the other hand, entropic methods are designed for the optimal manipulation of information.
